# Surface Functionalization of Anatase Nanopowders with Stearic Acid by Ball Milling

**DOI:** 10.3390/molecules31193430

**Published:** 2026-09-26

**Authors:** Maria Carta, Marta Cappai, Federico Cuccu, Giorgio Pia, Francesco Delogu

**Affiliations:** 1 Centre of Colloid and Surface Science (CSGI) Research Unit, c/o Department of Mechanical, Chemical and Materials Engineering, University of Cagliari, Via Marengo 2, 09123 Cagliari, Italy; mariacartabs@gmail.com (M.C.); federicocuccu50@gmail.com (F.C.); giorgio.pia@unica.it (G.P.); 2 Department of Mechanical, Chemical and Materials Engineering, University of Cagliari, Via Marengo 2, 09123 Cagliari, Italy; marta.cappai@unica.it

**Keywords:** powder processing, mechanical milling, kinetics, surface functionalization

## Abstract

This contribution is focused on the functionalization of the surface of anatase nanopowders following their mechanical processing by ball milling in the presence of stearic acid. We performed ball milling experiments using a ball mill that makes the reactor undergo three-dimensional oscillations, which result in an effective stirring of the powder mixture, employing a single milling ball. We changed the milling intensity by changing the ball mass. Analysis of the Fourier-transform infrared spectra suggests that surface functionalization occurs only within a relatively narrow range of milling intensity. Within such a range, we studied in great detail the kinetics of the surface modification. We also developed a kinetic model to interpret the experimental data and deepen the insight into the physical and chemical processes occurring on the microscopic scale.

## 1. Introduction

Titanium dioxide (TiO_2_) consists of Ti(IV) cations and oxide anions bonded to six oxide anions and three Ti(IV) cations, respectively. The network of chemical bonds gives rise to octahedral geometry with a Ti(IV) cation at the center. Differences in how octahedra are arranged and connected determine the existence of different TiO_2_ polymorphs [1,2,3,4,5,6].

Anatase, brookite and rutile are the three primary crystalline forms of TiO_2_ [2,7,8,9,10]. Rutile has the most compact crystalline structure, with slightly distorted octahedra sharing edges to form straight chains in a tetragonal symmetry. Octahedra distortion increases in anatase that, although maintaining a tetragonal symmetry, consists of winding chains of octahedra sharing four edges. In contrast, brookite crystals have orthorhombic symmetry, with six different Ti-O bond lengths and octahedra sharing only three edges [2,6,9,11,12,13,14].

The different crystalline structures exhibit different thermodynamic stability [15,16]. In particular, anatase and brookite are metastable phases that transform irreversibly in the rutile, the stable polymorph at room temperature and pressure, upon heating via a rearrangement of the octahedra network [6,16,17,18]. The bandgap is also affected by the crystalline structure, and it increases from about 3.0 eV to 3.2 eV and 3.4 eV in the sequence from rutile to anatase and brookite [19,20,21].

Such wide bandgaps allow the absorption of ultraviolet radiation and the corresponding generation of reactive electron–hole pairs that can cause the oxidation and reduction of chemical species adsorbed on TiO_2_ surfaces [6,16,22]. Combined with the remarkable chemical stability, this photocatalytic activity makes TiO_2_ one of the most versatile n-type semiconductor materials with broad application in water splitting, photovoltaic cells and the degradation of organic pollutants [22,23,24]. Other applications include structural ceramics, optical and corrosion-resistant coatings, gas sensing, electronics and white pigments [25,26,27,28,29].

In several contexts, the control of surface properties is a necessary condition to enable the successful use of TiO_2_ [5,30,31]. Valuable methods utilize surfactants to stabilize TiO_2_ particles in solution, taking advantage of steric and electrostatic effects to contrast attractive Van der Waals forces [5]. An interesting alternative to surface adsorption processes is the chemical modification of TiO_2_ surfaces, which ensures the direct chemical bonding of molecular species to the surface and makes the removal of the surface layer definitely more difficult [3,22,23].

So far, the chemical modification of TiO_2_ surfaces has been mostly pursued by using solution methods, which often have important drawbacks in the techno-economic and environmental costs [22,23,30,32,33]. More appealing is the surface functionalization of TiO_2_ powder particles in the absence of solvent phases [30,32,33,34,35]. It is not only more practical but also scalable to the industrial level [32,36,37]. In this respect, the mechanical processing of powders by ball milling (BM) can play a major role [36,38].

BM is a relatively simple mechanical processing method based on the effective deformation and fracture of powder particles that are compressed during the impacts between milling tools [39,40,41,42,43,44]. It can boast a long tradition in the fields of mineral refinement and materials science, and it is currently relevant to forefront research in mechanochemistry [41,45,46,47,48]. Concerning the different polymorphs of TiO_2_, BM has already been successfully utilized to reduce their particle size and increase their strain content, obtain nanocrystalline phases, induce their interconversion and even functionalize their surfaces [49,50,51].

While the overall picture suggests that the surface modification by BM has significant potential, and not limited to TiO_2_, it is undeniable that the available literature suffers from contradictory reports [10,52,53,54]. Any attempt of providing a deeper insight into the experimental evidence inevitably clashes with the remarkable uncertainties affecting the data collected and the lack of systematicity in investigating how BM conditions are reflected on the surface functionalization process [53,54,55,56]. The lack of information on the kinetic features of the surface functionalization of TiO_2_ by BM is a direct consequence [54,57].

In this work, we investigate in detail the surface functionalization of anatase powders induced by BM in the presence of octadecanoic acid, commonly known as stearic acid. This is a carboxylic acid that can interact with the TiO_2_ surfaces via different coordination configurations, including monodentate, bidentate bridging, and bidentate chelating modes [58,59]. It has already been used to modify the surface of TiO_2_ nanoparticles to enhance their hydrophobic character and facilitate their dispersion in polymer matrices or improve their interfacial adhesion properties [60,61]. An extensive experimental campaign allows us to clarify the main features of the process and the role played by the mechanical processing conditions. We show that stable association of stearic acid with the TiO_2_ powder, resistant to washing, can be reached only within a relatively narrow range of BM intensity. Within such a range, the discontinuous and statistical nature of BM results in significant fluctuations of the measured degree of surface functionalization. Nevertheless, the kinetics of the surface modification process can be satisfactorily characterized provided that the measurements involve a statistically relevant number of samples, which paves the way to a sound interpretation of the kinetic curve based on a kinetic model that takes into account the fundamental aspects of BM.

## 2. Results

**Figure 1:**
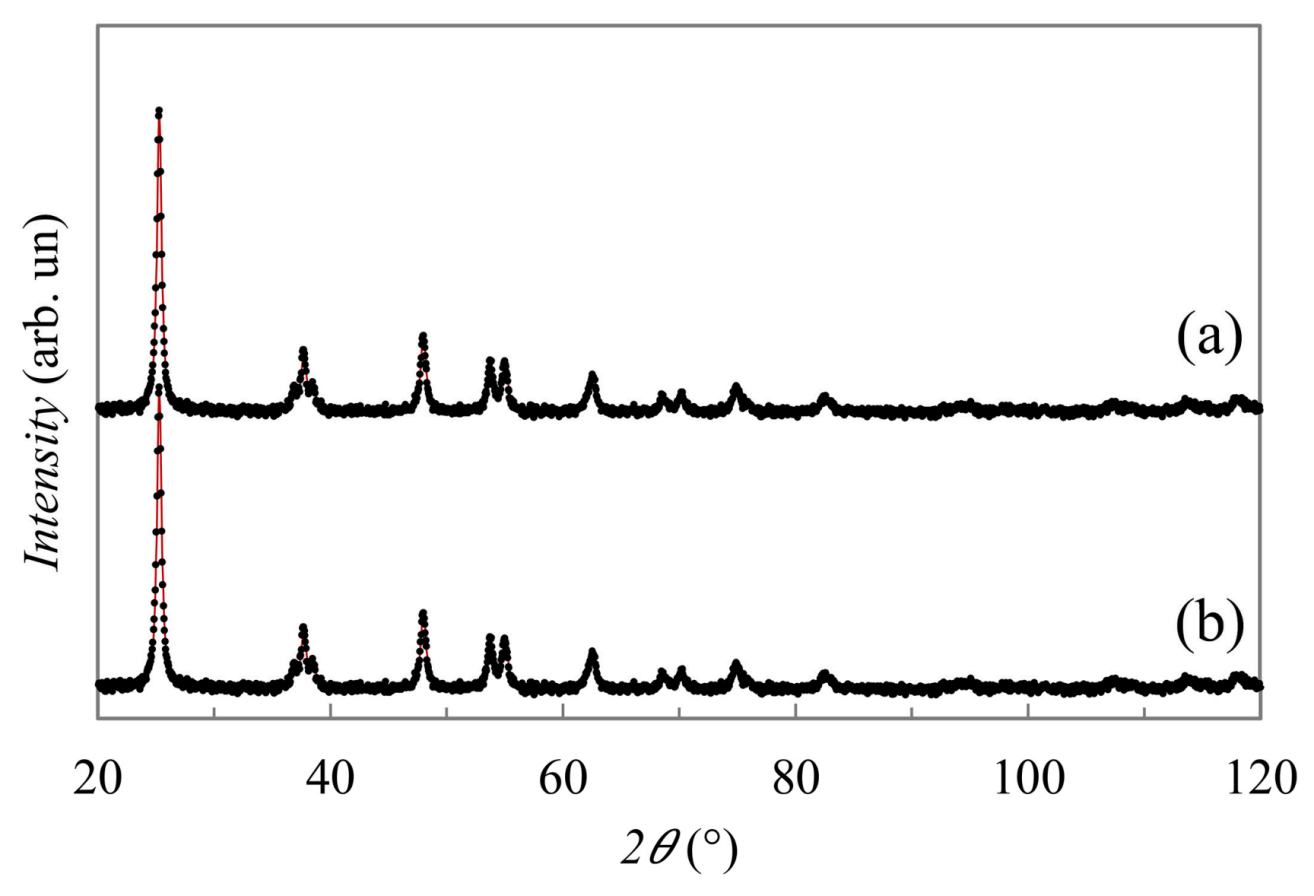
XRD patterns of pristine anatase nanopowders (**a**) and anatase nanopowders subjected to BM for 2 h (**b**). The comparison is representative of the whole set of ball masses investigated, since the patterns collected under the different conditions substantially overlap with each other. The red line represents the calculated pattern obtained from Rietveld refinement of the experimental XRD data. The anatase phase was identified using COD entry 5000223.

In principle, the mechanical processing of anatase powders by BM has the potential of inducing the transformation of anatase into rutile, with the so-called TiO_2_ II phase representing an intermediate between the two. For the objectives of the present study, this is highly undesirable. For this reason, we performed systematic X-ray diffraction analyses (XRDs) to verify the stability of the anatase under the selected BM conditions. Representative XRD patterns are shown in Figure 1.

It can be seen that the powder samples analyzed after relatively long BM times in the presence of a single stainless-steel ball of increasing mass exhibit the same XRD profile as the pristine anatase nanopowders. The different XRD patterns substantially overlap with each other regardless of the intensity of the mechanical processing. Therefore, we can be quite sure that the BM is unable to change their structure also when anatase nanopowders are mechanically processed in the presence of stearic acid. As a matter of fact, this compound has a waxy consistency that softens the effects of individual impacts. In particular, it reduces the impact elasticity, so that the ball transfers to the powder compressed during the impacts a fraction of its initial kinetic energy, equal to 0.9 and even more. At the same time, most of the mechanical energy that is transferred to powders is absorbed by stearic acid, which is the deformable component, while we can expect minor effects for hard anatase nanopowders.

**Figure 2:**
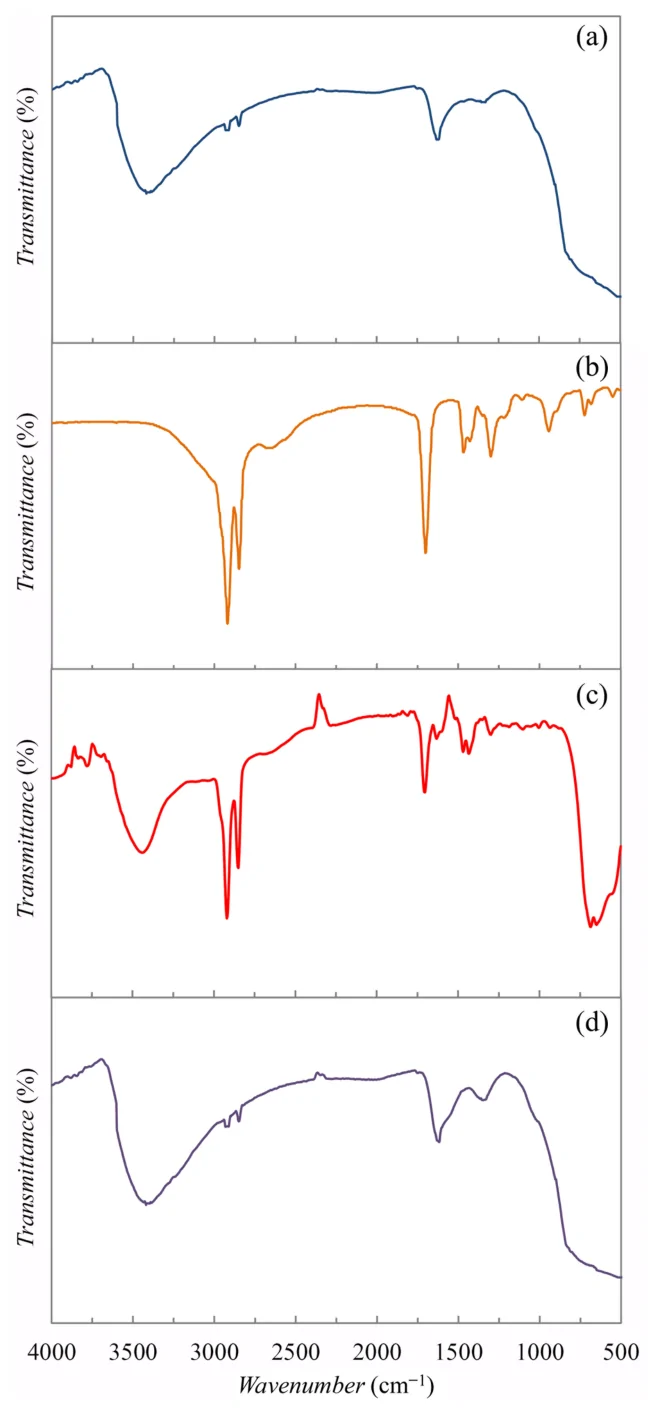
FTIR spectra of (**a**) pristine anatase nanopowders, (**b**) pristine stearic acid, (**c**) powder mixtures of anatase nanopowders and stearic acid subjected to BM for 40 min with a ball mass of 4.0 g, corresponding to an impact energy of about 0.032 J, and subsequently washed, and (**d**) powder mixtures of anatase nanopowders and stearic acid subjected to BM for 40 min with a ball mass of 5.0 g, corresponding to an impact energy of about 0.039 J, and subsequently washed.

Fourier-transform infrared spectroscopy (FTIR) provides information on the stearic acid associated with the anatase nanopowders, and on its interactions with the TiO_2_ surface. It also allows us to distinguish between the range of BM intensities in which surface functionalization occurs and those in which no detectable functionalization is observed. The FTIR spectra of the pristine anatase nanopowders, stearic acid, and the powders subjected to BM under functionalizing and non-functionalizing conditions are shown in Figure 2.

Pristine anatase exhibits an FTIR spectrum characterized by an intense broad band at wavenumbers approximately between 600 and 800 cm^−1^ that can be ascribed to the vibrations of the Ti-O and Ti-O-Ti chemical bonds [62,63]. The only other features detected are a broad band centered at about 3420 cm^−1^ and a band at about 1630 cm^−1^, which are ascribed to the stretching and bending vibrations of adsorbed water. A very similar spectrum is obtained for powders subjected to BM under non-functionalizing conditions, indicating that no detectable, or only a negligible, amount of stearic acid remains associated with the powder. In contrast, the powders that have undergone mechanical processing display peaks that can be attributed to vibrations of the chemical bonds characteristic of the stearic acid [64]. We observe a distinctive peak at about 720 cm^−1^ for the rocking of long aliphatic chains, relatively sharp peaks in the range between 1462 and 1470 cm^−1^ for the bending of methylene moieties, a sharp intense peak at about 1705 cm^−1^ for the stretching of the carbonyl group, and relatively intense peaks for the symmetric and asymmetric stretching of the methylene moieties respectively around 2850 and 2920 cm^−1^. The appearance of these stearic-acid-related features under functionalizing conditions, together with additional spectral changes in the carboxylate region, is consistent with the formation of strongly bound stearic-acid-derived surface species.

The intensity of the FTIR spectrum generated by the stearic acid is so high as to hide the one characteristic of the anatase. It is worth noting, however, that the washes carried out immediately after the mechanical processing are able to remove all the stearic acid in excess, leaving only the molecules that have a direct chemical bond or a very strong interaction with the anatase surface. The only way to remove the stearic acid that functionalizes the anatase surface is to heat the processed powder to relatively high temperatures.

The systematic change in the ball mass, which corresponds to a systematic change in the impact energy, and the subsequent FTIR analyses reveal that the mechanical processing by BM is not always capable of inducing the functionalization of the anatase surfaces. We started our investigation by simply agitating the powder mixtures of anatase and stearic acid. To this end, we placed the powder mixture inside the reactor without the milling ball, fixed it to the clamp assembly of the Spex Mixer/Mill 8000 and operated the ball mill for relatively long times. The FTIR spectra recorded at the end of the washing and drying procedures almost perfectly overlap with the FTIR spectrum of pristine anatase nanopowders, thus providing no detectable evidence of chemical modification of the anatase surfaces.

**Figure 3:**
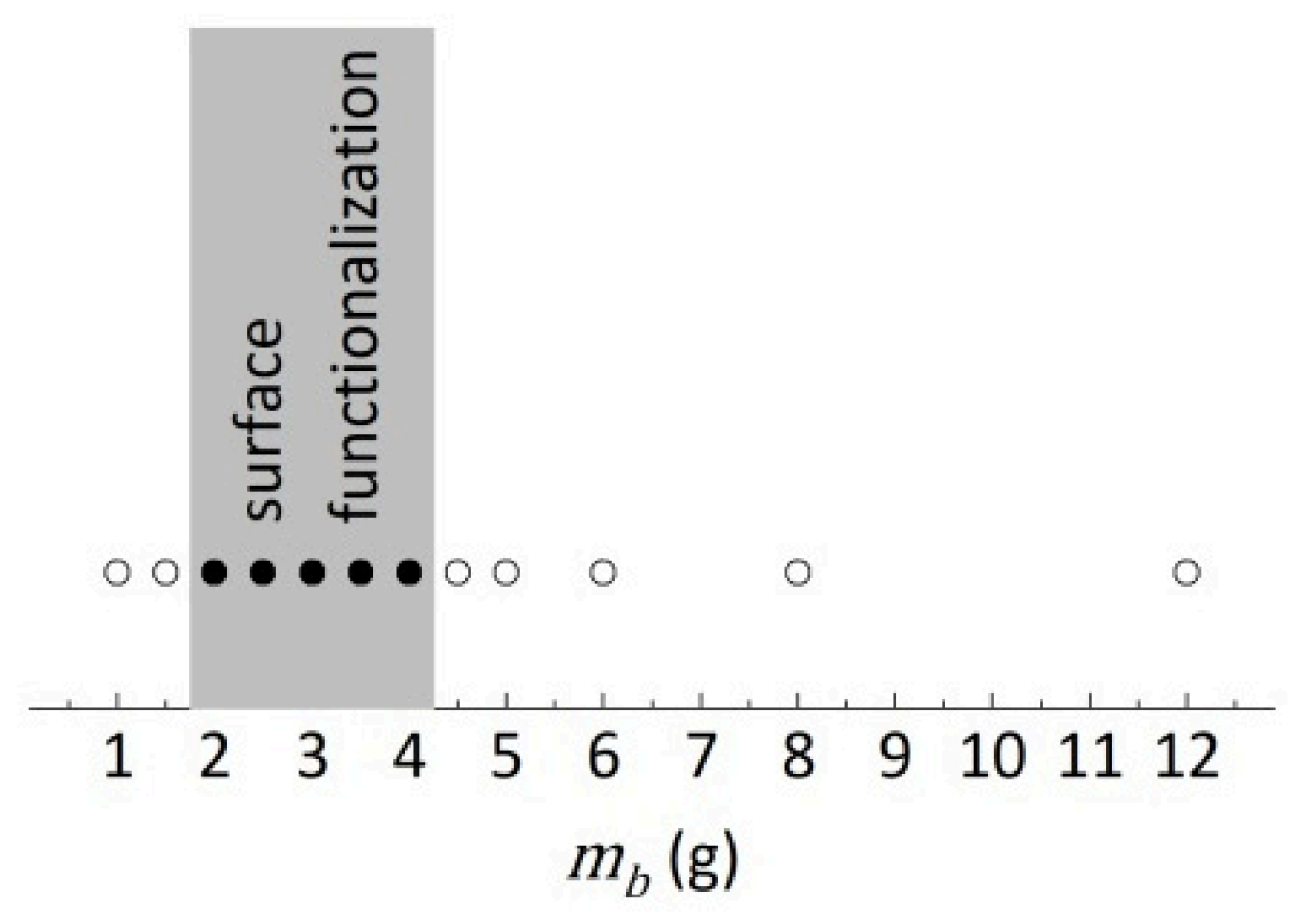
Qualitative results of BM experiments as a function of the ball mass, $m_{b}$. Circles are used to indicate successful (●) and unsuccessful (○) surface functionalization. For convenience, we added the shaded area encompassing the $m_{b}$ values for which surface functionalization occurred.

Then, we performed BM experiments using single stainless-steel balls of mass from 1.0 to 12.0 g. The results are summarized in Figure 3.

It can be seen that we obtained evidence of surface functionalization only for powder mixtures subjected to BM using a ball mass, $m_{b}$, in the range from 2.0 to 4.0 g, which corresponds to a range of impact energy, E, between 0.016 and 0.032 J. Outside this range, the FTIR spectra of processed powders exhibit no characteristic peak that can be associated with the presence of stearic acid.

To a first approximation, the missed functionalization of anatase surfaces at low impact energies can be generically explained with the hypothesis that the chemical reactivity of anatase surfaces needs some enhancement to eventually allow the stable interaction of the molecules of the stearic acid in contact with the anatase nanoparticles. For instance, we can reasonably hypothesize that impacts must induce effective frictional processes at the surface of the anatase nanoparticles to promote a stable interaction with stearic acid, which can happen only if the impact energy is above a certain threshold.

More surprising is the evidence that the surface functionalization no longer occurs above a higher threshold in impact energy. To investigate this issue, we subjected to BM, in two distinct BM experiments in the presence of a single ball with mass equal to 8.0 g and 12.0 g, suitable amounts of powder mixtures that, subjected to BM with a 4.0 g ball, contain anatase nanoparticles with a functionalized surface. After the BM at impact energies equal to 0.057 and 0.078 J, the FTIR spectra no longer provide evidence that the surface of anatase nanoparticles is functionalized. The FTIR spectra overlap almost perfectly with that of pristine anatase nanopowders. Therefore, we can only infer that, when the impact energy is too high, the processing conditions are not compatible with the stable association of the stearic acid with the anatase surfaces. Even more, we have evidence that impact energies outside the optimal range induce the degradation of functionalized surfaces and the removal of the stearic acid from the anatase surfaces. We are tempted to justify the latter observation with the temperature rise that individual impacts can induce in small regions of the powder compressed during the impact, but we have no real evidence that can support this hypothesis.

**Figure 4:**
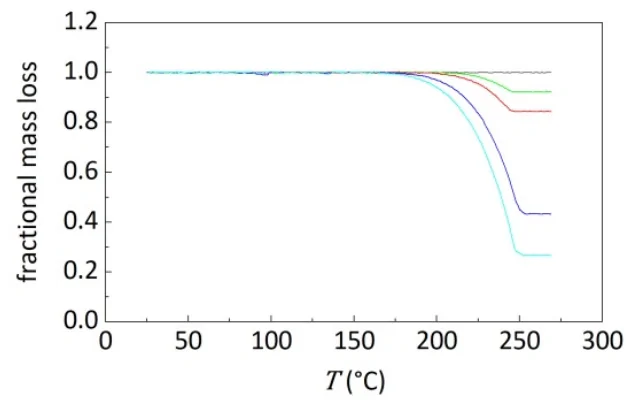
TGA thermograms showing the fractional mass loss as a function of temperature, T. Data refer to powder mixtures subjected to BM at an impact energy of about 0.032 J. From top to bottom, thermograms refer to pristine anatase nanopowders (grey line) and to powder mixtures subjected to BM for 5 (green), 15 (red), 30 (blue) and 40 (cyan) min respectively.

Once we identified the impact energy range that allows the modification of the anatase surfaces, we focused on the kinetics of the functionalization process driven by BM. In this respect, thermogravimetric analysis (TGA) provided valuable datasets. A typical sequence of thermograms is shown in Figure 4.

For pristine anatase nanopowders, the thermograms simply consist of a horizontal line, which indicates that no mass loss takes place. This is expected, given that the anatase nanopowders were dried, stored and handled invariably under Ar atmosphere.

The thermal behavior of the processed powder mixtures is completely different. We observe significant mass losses, the extent of which depends on the time, t, during which the powder mixtures have experienced BM. In particular, the longer the time of mechanical processing, the larger the mass loss. The mass loss seems to begin at temperatures that decrease as the time t of mechanical processing increases. In contrast, the latter has no detectable effect on the temperature at which the mass loss reaches completion, which is around 250 °C.

**Figure 5:**
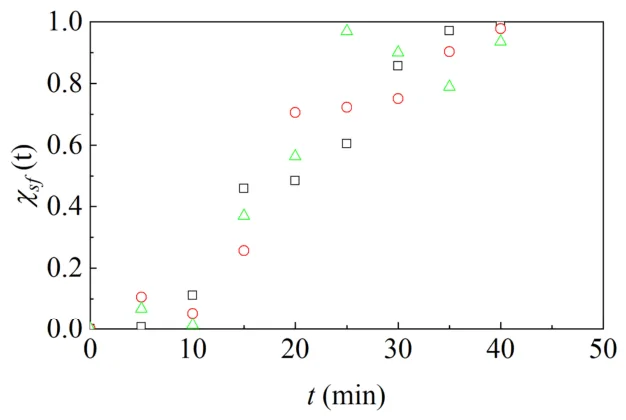
The normalized degree of surface functionalization, $\chi_{sf}(t)$, as a function of time, t. Data refer to powder mixtures subjected to BM at an impact energy of about 0.032 J. Different symbols refer to three independent sets of measurements.

The mass losses, normalized to the maximum mass loss detected, provide a suitable quantity to describe the kinetics of surface functionalization. Under the investigated conditions, the maximum mass loss corresponds to the maximum amount of stearic acid stably associated with the anatase surface under the investigated conditions. This fraction is not removed by the washing and subsequent drying procedures and is independently evidenced by the characteristic stearic acid-related bonds observed in the FTIR spectra. Representative datasets of the resulting normalized degree of surface functionalization, $\chi_{sf}(t)$, defined as the ratio between the mass loss measured at a given processing time and the maximum mass loss measured, are plotted in Figure 5 as a function of the BM time, t, with increas interpreted as showing an increase in the functionalized fraction with increasing t. However, the data are significantly scattered, which makes the kinetic curve extremely noisy and quite undetermined in view of a possible mathematical description.

In this regard, it is worth noting that each dataset consists of a sequence of single measurements from processed powders sampled at increasing times of BM. The three datasets taken into consideration in Figure 5 represent the result of BM experiments performed under the same identical mechanical processing conditions. It appears extremely clear that we are far from observing any possible reproducibility of the mass losses induced by the heating process underlying TGA. It is clear as well that the uncertainties inherently associated with such measurements are quite large, undermining the reliable identification of the surface functionalization kinetics.

Note that this issue does not stem from mistakes in the design of BM experiments or errors in the experimental methods. It is a problem caused by the fact that TGA measurements are performed using tiny samples of the processed powder mixture, with a mass equal to 5 mg which are not representative of the total mass of processed powders, equal to 8 g. Samples are too small, and the isolated measurement of mass loss on such samples inevitably brings out the inhomogeneity of the processed powders, which is a clear consequence of the discontinuous and statistical nature of the mechanical processing of powders by BM.

**Figure 6:**
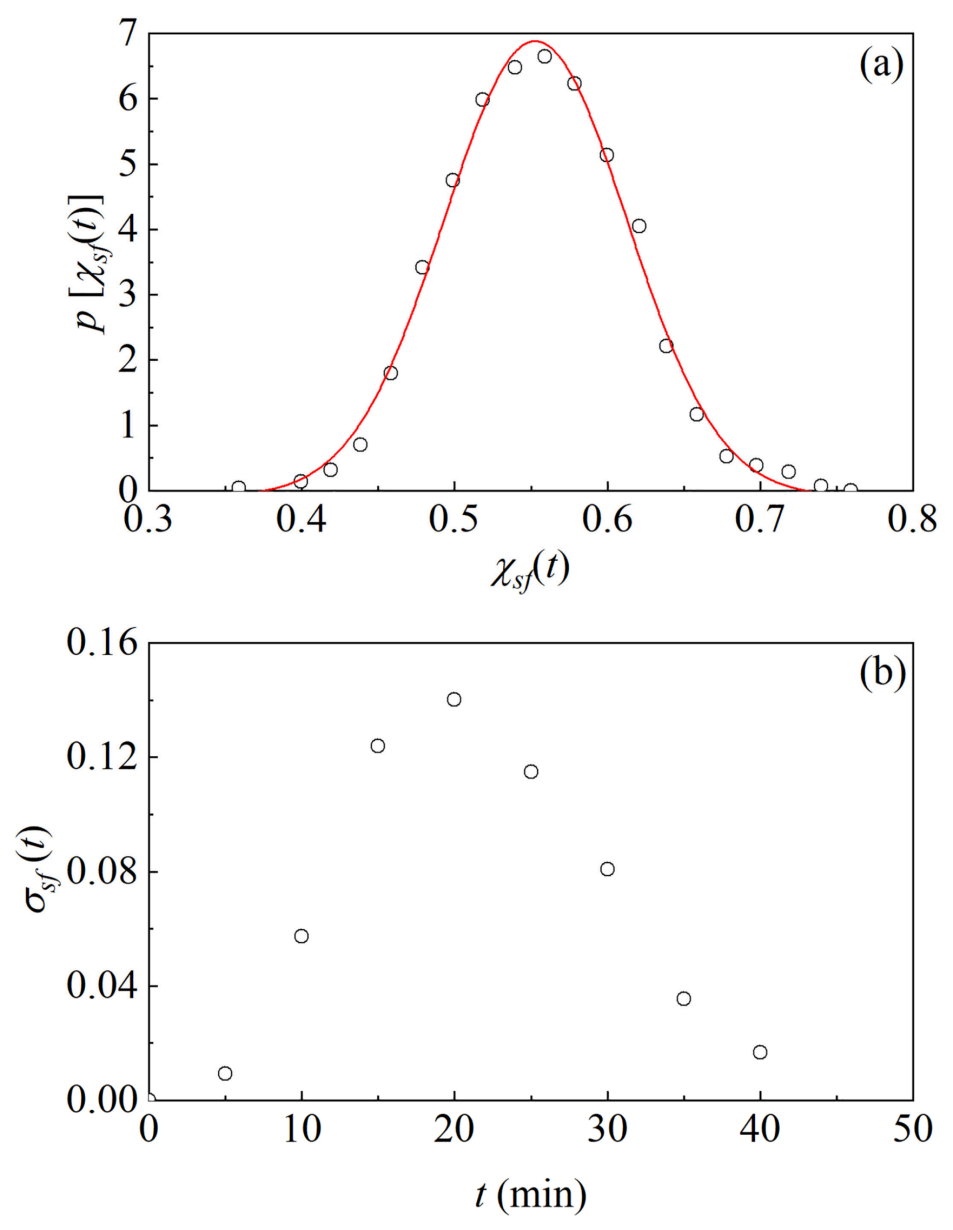
(**a**) The statistical distribution of the normalized degree of surface functionalization, $\chi_{sf}(t)$, as a function of time, t. The red line represents the corresponding fit. Data refer to powder mixtures subjected to BM at an impact energy of about 0.032 J. (**b**) The standard deviation, $\sigma_{sf}(t)$, of the statistical distribution of the normalized degree of surface functionalization, $\chi_{sf}(t)$. Data refer to powder mixtures subjected to BM for 20 min at an impact energy of about 0.032 J.

The problem can be solved by analyzing a statistically representative number of samples, and as we have done for BM experiments performed using a single ball of 4.0 g. Specifically, we have performed TGA measurements on 60 different samples of 5 mg and evaluated the statistical distribution of the mass losses measured. The results obtained for a single time of mechanical processing, equal to 20 min, are shown in Figure 6a. It can be seen that the measurements are distributed according to a Gaussian curve centered on a $\chi_{sf}(t)$ value of about 0.553 and characterized by a standard deviation, $\sigma_{sf}(t)$, of about 0.140.

We have repeated the same procedure for all the different times of mechanical processing investigated. As shown in Figure 6b, the standard deviation $\sigma_{sf}(t)$ changes significantly with the time t, reaching a maximum approximately after 20 min of BM. We believe that this change highlights the underlying change in the homogeneity of the processed powder mixtures.

**Figure 7:**
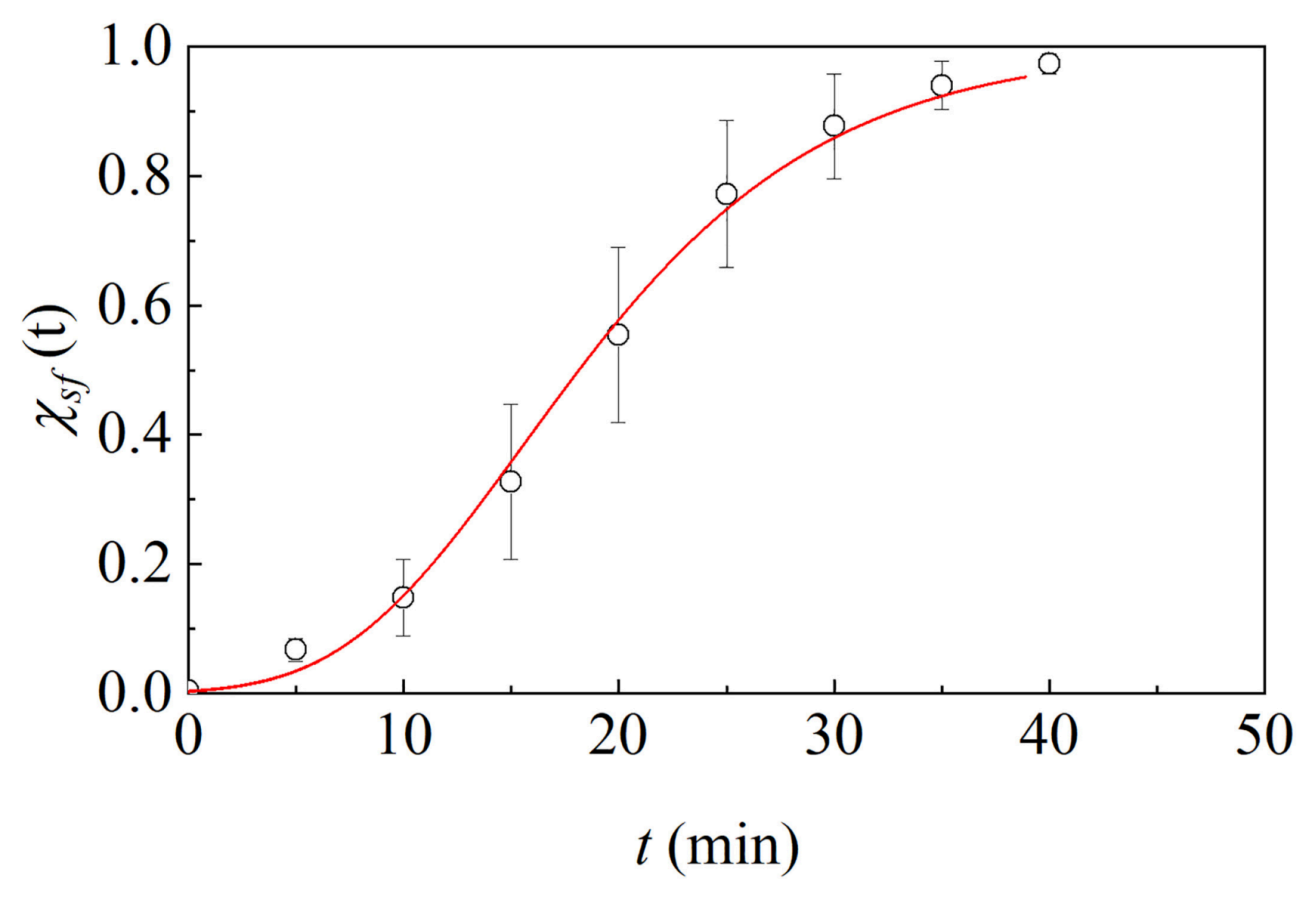
The normalized degree of surface functionalization, $\chi_{sf}(t)$, as a function of time, t. Error bars correspond to the standard deviation, $\sigma_{sf}(t)$, of the statistical distribution of the normalized degree of surface functionalization, $\chi_{sf}(t)$. Data refer to powder mixtures subjected to BM at an impact energy of about 0.032 J. Best-fitted curve (red) is also shown.

The refined kinetic dataset eventually obtained is shown in Figure 7. Data are arranged according to a smooth sigmoidal curve, which suggests that the surface functionalization process can be considered complete after about 50 min of BM.

## 3. Discussion

The experimental findings presented so far provide a clear view of how much the evaluation of kinetic quantities can be affected by the intrinsic inhomogeneity of the mechanical processing by BM. The use of samples that are far from being representative of the entire mass of processed powder can result in misleading conclusions or even completely hide a phenomenon from the experimental observation. For sure, it prevents a quantitative interpretation of the experimental findings and, thus, a proper understanding of the underlying mechanisms and processes. Therefore, it is of paramount importance to take into account the impact of the statistical factors on the results of the investigation carried out, an aspect that is too often overlooked.

While the considerations above are valid in any case potentially affected by statistical factors, there are at least three main points, specific to the subject of this work, to which it is worth drawing attention.

The first point concerns the Gaussian distribution of the $\chi_{sf}(t)$ estimates obtained by TGA. As a matter of fact, the Gaussian distribution can be regarded as a reliable indication of randomness in sampling. More specifically, our sampling procedure satisfies the so-called Central Limit Theorem, according to which the distribution of sample averages from any statistical population tends to approximate a Gaussian distribution as the number of samples increases, provided that the samples considered are independent and identically distributed. This means that our sampling procedure was not biased by factors related to the handling of processed powders or their properties. Thus, although the processed powders are far from being compositionally homogeneous, the way we sample them is homogeneous.

The second point to consider is the shape of the kinetic curve. The reason is that the sigmoidal shape is quite unexpected. To a first approximation, the surface functionalization process involves the activated surface of anatase nanopowders and the surrounding molecules of stearic acid. Whenever and wherever the anatase surface is prone to reaction, new chemical bonds with the molecules of stearic acid are formed. Under these circumstances, surface functionalization proceeds in proportion to the availability of unreacted surface. Given that the chemical modification of the surface with stearic acid reduces the anatase surface available for further reaction, we should expect to observe a monotonic decrease in the reaction rate with the coverage of the surface and, then, a kinetic curve of exponential character that tends to reach the final value asymptotically. Definitely, the kinetic curve we observe is sigmoidal, which raises questions about the details of the chemical interaction between the anatase surface and the stearic acid.

We refer immediately to the interfacial interactions involved in the surface functionalization process because we think that other possible factors can be ruled out. For instance, we can exclude that the mechanical processing by BM induces a change in the nature of the anatase surfaces or induce a comminution of the anatase nanopowders. BM leaves anatase nanopowders substantially unaffected, except for some sort of surface activation during the impacts, which we are unable to characterize and define, without which the chemical modification of the anatase surface no longer occurs. Along the same line, no change in the properties of the stearic acid can be invoked. Similarly, we can reasonably expect that impacts result in the same dynamic compaction conditions throughout the entire mechanical processing.

These considerations bring us directly to the third point of interest, namely the temperature at which TGA induces the mass loss in powder mixture processed by BM for different times. We have already pointed out that the shorter the time of mechanical processing, the higher the temperature at which mass loss starts. We interpret this experimental observation as evidence of the fact that the molecules of stearic acid that have reacted earlier are more difficult to remove from the anatase surface than the others that are progressively added. The existence of sites of different reactivity on the surface of anatase nanoparticles represents the most probable rationale for such behavior. The most reactive sites can be expected to react first with the molecules of stearic acid and form most stable surface species, including coordinate carboxylate species that can be ascribed to the weak peaks around 1500 in the FTIR spectra, which immediately translate into higher temperatures to break them. Therefore, it is reasonable to think that surface reactivity modulates the chemical reaction with the stearic acid.

Having said all of this, we believe that the kinetic curve we have built in Figure 7 contains enough information to shed light on the open questions regarding the sigmoidal character of the surface functionalization kinetics and its possible dependence on the existence of surface sites characterized by different reactivity. A necessary condition to proceed successfully in this direction is the suitable description of the effects of individual impacts on the chemical modification of the anatase surfaces, which requires taking into account the discontinuous and statistical nature of the mechanical processing by BM. To this aim, we refer to a kinetic model developed expressly to describe the kinetics of mechanically activated transformations.

Described in detail elsewhere [65,66,67,68], the kinetic model relates the global kinetics observed on the macroscopic scale to the statistics underlying the mechanical processing by BM and the local kinetics governing the phase transformation at the length scale of powder particles involved in individual impacts. Here, it is worth reminding that the model is kept as simple as possible because a more detailed microscopic description would require a large number of quantities and parameters that cannot be independently determined from the available experimental data.

First of all, we assume that, when a small volume $v^{*}$ of powder is affected by mechanical stresses due to an impact, the stearic acid undergoes flow deformation. Time by time, deformation involves the aggregates of anatase nanoparticles that inevitably form in nanopowders because of adhesion forces of different origin. This allows the generation of a new interface area between anatase and stearic acid. Repeated deformation events eventually result in the intimate mixing of the two components of the powder mixture, bringing the surface of anatase nanoparticles fully in contact with the stearic acid. For convenience, we refer to these deformation events with the name of critical loading conditions (CLCs).

The model does not attempt to describe in detail the microscopic mechanisms by which this interfacial area is generated. Rather, it assumes that the overall increase in the anatase surface area available to interact with the stearic acid $S_{i}$ in volumes $v^{*}$ after i CLCs can be described phenomenologically as an autocatalytic process and are equal to

(1) $$S_{i}=S_{0}S_{fin}/\left[S_{0}+\left(S_{fin}-S_{0}\right)\exp\left(-ri\right)\right],$$

where $S_{0}$ and $S_{fin}$ are the initial and final surface areas available for chemical reaction, respectively, and r is the rate of surface area increase.

It is worth noting that Equation (1) describes only the generation of new surface area available to chemical modification. However, the availability of surface area does not necessarily result in the surface functionalization with the molecules of stearic acid. Other energetic and steric conditions must be fulfilled for stable surface functionalization to occur. These can be taken into account on a phenomenological basis by defining an interaction probability parameter Π that can take values between 0 and 1 and represents the probability that stearic acid molecules establish stable interactions with the anatase surface.

Under the simplifying assumption that all the anatase nanoparticles have the same size and, then, the same surface area $s_{anp}$, the maximum surface area available for stable interaction with stearic acid, $S_{max}$, must be equal to the product $N_{anp}\;s_{anp}$, where $N_{anp}$ is the total number of anatase nanoparticles in the powder mixture. For simplicity, we assume that the plateau in functionalization kinetics corresponds to the limiting condition in which repeated CLCs have made the powder progressively accessible to processing, so that the final surface area available to chemical reaction, $S_{fin}$, corresponds to the maximum surface that can be generated between the molecules of stearic acid and the anatase nanopowders. We can calculate the maximum number of molecules of stearic acid that can functionalize the anatase surface, $N_{sa,max}$, by the product $N_{anp}\;s_{anp}/s_{sa}$, where $s_{sa}$ represents the surface area occupied by a single molecule of stearic acid interacting with the anatase surface. This quantity is known to correspond to about 0.2 nm^2^ per molecule.

We can expect that the number of molecules of stearic acid that react with the anatase surface in a volume $v^{*}$ subjected to CLCs during an impact, ${∆N}_{sa}$, is proportional to the surface area $S_{i}$. However, we have to consider that not all the surface area $S_{i}$ is available for reaction, but only the portion not yet occupied by molecules of stearic acid interacting with the surface. The portion occupied by stearic acid is equal to $N_{sa,i}\;s_{sa}$, where $N_{sa,i}$ is the number of molecules interacting with the anatase surface after the volume $v^{*}$ has experienced i CLCs. In addition, we also suppose that the degree of surface occupation can also affect the interaction probability Π. In this regard, we can expect, at least in principle, that the reaction between the anatase surface and the stearic molecules becomes increasingly disfavoured as the surface functionalization increases. Accordingly, it is reasonable to expect that the interaction probability decreases. For this reason, we implicitly account for its possible variation by denoting it with $\Pi_{i}$. Therefore, we can write that

(2) $${∆N}_{sa,i+1}=\Pi_{i}\left(S_{i}-N_{sa,i}\;s_{sa}\right),$$

which means that $N_{sa,i+1}$ is equal to $N_{sa,i}+{∆N}_{sa,i+1}$. The corresponding normalized degree of surface functionalization, $\chi_{sf,i}$, can be calculated by multiplying $N_{sa,i+1}$ by $s_{sa}/S_{fin}$.

Equation (2) can be solved numerically, thus providing a description of the surface functionalization kinetics in volumes $v^{*}$ as a function of the number i of CLCs experienced. To move from the local kinetics, described by $\chi_{sf,i}$, to the global one, referring to $\chi_{sf}\left(t\right)$, we need to consider the statistics underlying the involvement of volumes $v^{*}$ in CLCs.

In this regard, it is worth noting that, in the presence of effective stirring processes of powders and ball, the powders inside the reactor always keep a high degree of mixing [69]. The complexity of the milling dynamics is such that it is impossible to predict which powder particles will be compressed at impact. Accordingly, we make the simplifying assumption that any given impact affects an amount of powder randomly selected from the entire powder charge.

Such assumption implies that volumes $v^{*}$ are also affected by CLCs stochastically. It follows that the total volume V of powder inside the reactor consists of volumes $v^{*}$ that have been affected by CLCs different times. We can write that the volume fraction of powder that experiences CLCs during a single impact, κ, is equal to the ratio v/V, where v is the sum of the volumes $v^{*}$ affected by CLCs in a single impact. Furthermore, the volume fraction of powder that has undergone CLCs i times after m impacts can be expressed as

(3) $$\chi_{i}\left(m\right)=\left[{\left(\kappa m\right)}^{i}/i!\right]exp\left(-\kappa \;m\right).$$

The condition

(4) $$\sum_{i=0}^{\infty }\chi_{i}\left(m\right)=1$$

is always fulfilled.

The normalized degree of surface functionalization, $\chi_{sf}\left(m\right)$, after m impacts is given by the weighted sum

(5) $$\chi_{sf}\left(m\right)=\sum_{i=0}^{m}\chi_{i}\left(m\right)\chi_{sf,i}$$

**Figure 8:**
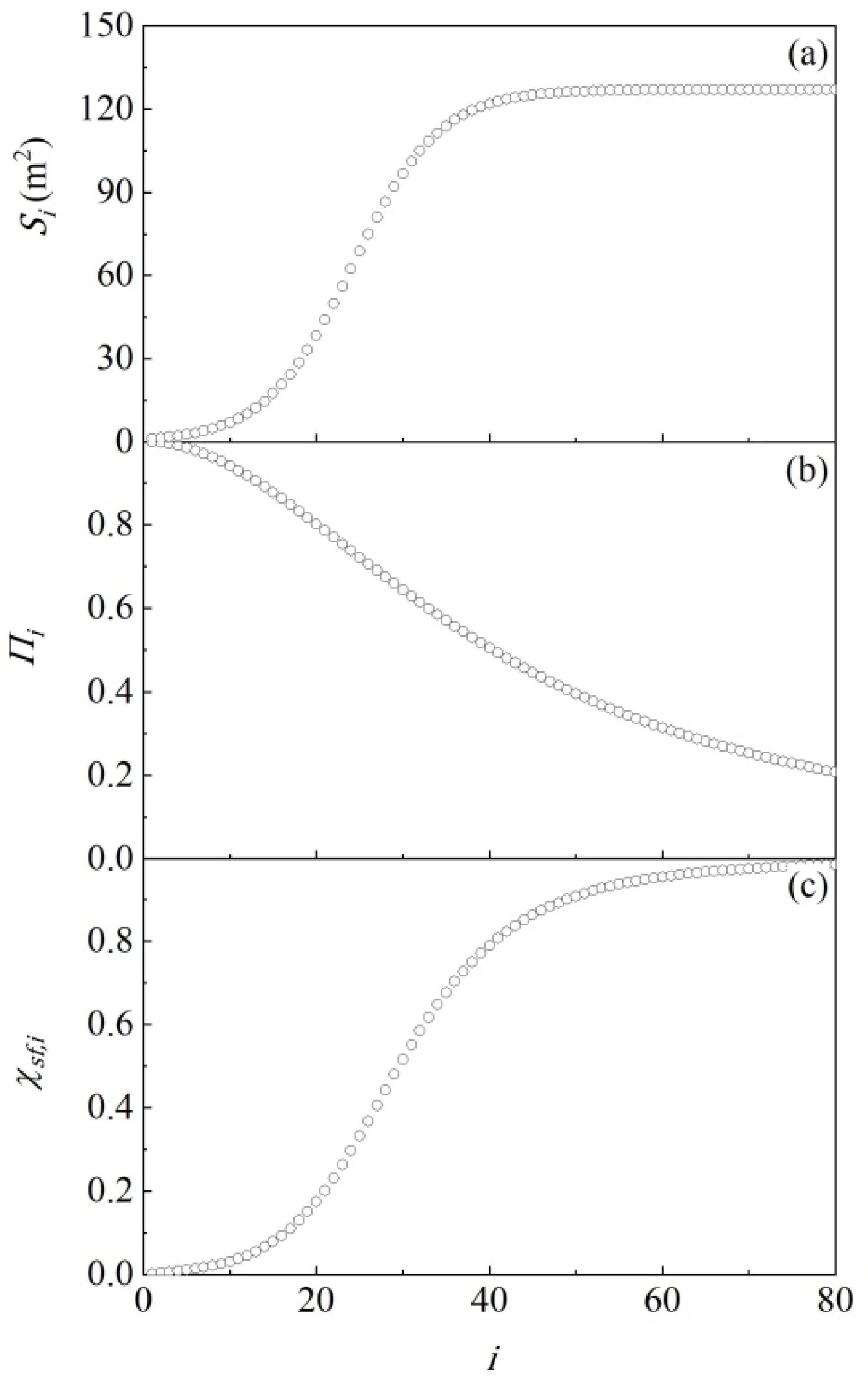
(**a**) The surface area of anatase nanopowders, $S_{i}$, (**b**) the reaction probability, $\Pi_{i}$, and (**c**) the normalized degree of surface functionalization, $\chi_{sf,i}$, in volumes $v^{*}$ as a function of the number i of CLCs experienced by the volumes $v^{*}$. Data were obtained by the best fitting procedure regarding powder mixtures subjected to BM at an impact energy of about 0.032 J. Circles represent the values calculated by the model.

Equation (5) is the kinetic equation that we use to best-fit the experimental data plotted in Figure 7. The use of a least-squares method to perform the best fit pointed out a satisfactory agreement between the model and the experimental dataset. The robustness of the fitting procedure was assessed by repeating the optimization from multiple initial parameter sets distributed over the physically meaningful parameter ranges. These ranges were identified by following rules that emerge from physical and mathematical considerations. For instance, the probability factor must range between 0 and 1. We assume that the final surface should be greater than or equal to the initial surface. The surface area generation rate must take values compatible with a progressive increase from $S_{0}$ to $S_{fin}$. Within the explored parameter space, the procedure consistently converged to essentially the same set of fitted parameters. No alternative solution providing a comparable goodness of fit was found, with the resulting fit characterized by a coefficient of determination R^2^ about 0.987. Therefore, there is only one fitting solution where the surface area of anatase nanopowders, $S_{i}$, the reaction probability, $\Pi_{i}$, and, then, the normalized degree of surface functionalization, $\chi_{sf,i}$, in volumes $v^{*}$, change with the number i of CLCs experienced by the volumes $v^{*}$, as shown in Figure 8.

The surface area of anatase nanopowders, $S_{i}$, exhibits an initial value $S_{0}$ of about 1 m^2^ and a final one $S_{fin}$ around 126 m^2^. This is absolutely comparable with the specific surface area of 61.4 m^2^ g^−1^ that can be expected for non-agglomerated anatase nanoparticles that have a characteristic size around 25 nm. Therefore, we can reasonably infer that, initially, agglomeration almost completely limits the surface functionalization process. The mechanical processing induces the progressive disaggregation of anatase nanoparticles until an approximately uniform dispersion in the mass of stearic acid is attained. The increase in surface area from $S_{0}$ to $S_{fin}$ requires that volumes $v^{*}$ experience 40 CLCs or more, which translates into a rate constant for surface area increase, r, equal to about 0.21.

Surface area is not the only quantity to change as a consequence of BM. The interaction probability, $\Pi_{i}$, also changes. In particular, it decreases smoothly from an initial value of about 1 to values as low as 0.2. We interpret the change as evidence that the reaction of the stearic acid with the anatase surfaces becomes increasingly hindered by the molecules bound to the surface. Recalling that thermograms indicate that the temperature at which mass losses occur increases with the time of mechanical processing, we tentatively explain the $\Pi_{i}$ change by hypothesizing that the surface of anatase nanopowders exhibits sites with different reactivity. However, we note that steric effects due to the presence of bonded molecules also have the potential to make the chemical modification of the anatase surface progressively more difficult. In both cases, we must expect a reaction probability $\Pi_{i}$ that becomes increasingly smaller in agreement with the results suggested by the best fitting.

According to Equation (2), the combination of surface area and reaction probability determines the normalized degree of surface functionalization, $\chi_{sf,i}$, in volumes $v^{*}$. The local kinetics are described by a smooth sigmoidal curve that suggests that surface functionalization in volumes $v^{*}$ reaches completion when they experience approximately 60 CLCs. This number is three orders of magnitude smaller than the total number of impacts experimentally incurred at the end of the surface functionalization process, which is approximately equal to 7 × 10^4^.

The huge difference between number of CLCs and number of impacts shows the relevance of statistics in shaping the results of the mechanical processing by BM. The best fitting procedure suggests that the fraction of powder mixture effectively processed during individual impacts, κ, is approximately equal to 9 × 10^−4^. Accordingly, only about 0.2 mg of powder is involved in the surface functionalization of anatase nanoparticles in a given impact. This is a tiny amount that makes it easy to understand how many impacts are needed to effectively work the whole amount of powder inside the reactor. It is inevitable that the overall surface functionalization process requires relatively long times of BM.

The most important consequence of the scenario depicted so far is that the kinetic curve obtained experimentally does not really provide a description of the functionalization kinetics characteristic of the chemical system at the microscopic scale. On the contrary, the kinetic data shown in Figure 7 result from the combination of three different factors, which are related to the statistics inherently underlying the mechanical processing of powders by BM, the rheological behavior of the powders affected by impacts time by time and the reactivity of the chemical substances that experience the CLCs during the impact [70]. The probability factor, which is the only one which gives information on the functionalization process, only emerges once the other factors are properly accounted for. It follows that only the data shown in Figure 8 shed light on the kinetics of the surface functionalization undergone by anatase nanopowders under BM conditions. The kinetic data plotted in Figure 7, which represent the experimental evidence that can be collected, can be misleading if not suitably interpreted within the framework of a kinetic model coherent with the inherent nature of the mechanical processing of powders by BM.

## 4. Materials and Methods

Experiments were performed using high-purity anatase nanopowder (Sigma Aldrich, St. Louis, MO, USA, <25 nm particle size, 99.7% trace metals basis) and stearic acid (Sigma Aldrich, >95%). The anatase nanopowder was placed in an oven kept at 100 °C and exposed to a flux of an Ar-H_2_ gaseous mixture with 10% of H_2_ for 12 h to remove undesired chemical species from the nanoparticle surface. At the end of the thermal treatment, the nanopowder was stored under Ar atmosphere with nitrogen, oxygen and a humidity content below 3 ppm.

To further activate the nanoparticle surfaces, the nanopowder was subjected to mild mechanical activation. To this end, it was placed in a hardened-steel cylindrical reactor of about 60 cm^3^ in volume, sealed under inert atmosphere, and clamped to the mechanical arm of a Spex Mixer/Mill 8000 ball mill (SPEX SamplePrep LLC, Metuchen, NJ, USA). The ball mill was operated for 2 h at 14.6 Hz to allow the stirring of nanopowders and the occurrence of frictional processes.

BM experiments aiming at the functionalization of nanoparticles were performed under the same conditions. We used 2 g of anatase nanopowder and 6 g of stearic acid. The mixture was manually mixed once placed inside the cylindrical reactor. Then, a single stainless-steel ball was added and the reactor sealed. We used balls of different sizes and masses to verify the effects of the impact energy on the surface functionalization process. The ball mill was interrupted every 5 min and the processed powder suitably sampled to monitor the surface modification.

We used X-ray diffraction (XRD) to characterize the anatase crystal structure and verify that no phase transformation occurs as a consequence of BM. XRD patterns were collected with a Rigaku D/Max diffractometer (Rigaku Corporation, Tokyo, Japan) equipped with a Cu *Kα* radiation source.

The chemical modification of the surface was monitored by thermogravimetric analysis (TGA). Suitable amounts of processed powders were washed with distilled water at 70 °C for 15 min under agitation, and the resulting aqueous solution was filtered. The procedure was repeated three times. Finally, the filtered powders were dried in an oven under Ar flux at 100 °C for 12 h. A mass of 5 mg of powder was accurately weighed and placed in an alumina crucible. The mass loss was measured using a Mettler Toledo thermal analysis system TGA 2. The sample was heated from 25 °C to 300 °C at a heating rate of 5 °C min^−1^ in an Ar atmosphere.

Fourier-transform infrared spectroscopy (FTIR) analyses were performed to verify the occurrence of the surface functionalization of the anatase surface and to identify the BM conditions under which it takes place. A Bruker Vertex 70 spectrometer (Bruker Optics GmbH & Co. KG, Ettlingen, Germany) was used. Samples were analyzed in a spectral range from 500 to 4000 cm^−1^.

The milling intensity was evaluated by estimating the impact frequency and velocity of the single milling ball. We used a method combining experimental measurements taking advantage of piezoelectric sensors and the numerical reconstruction of ball trajectories inside the reactor. In this way, we are able to satisfactorily evaluate the impact frequency, N, the impact elasticity, measured by a coefficient of restitution, e, and the impact velocity, $v_{imp}$. Accordingly, we can also calculate the impact energy, E, which we define as the mechanical energy transferred to powders. Specifically, E is equal to $m_{b}{\left(1-e\right)}^{2}v_{imp}^{2}/2$. The method is described in detail elsewhere [71]. Here, we simply provide the results of our investigation, summarized in Table 1.

## 5. Conclusions

We investigated the surface functionalization of anatase nanopowders subjected to mechanical processing by BM in the presence of stearic acid. Our experimental findings indicate that anatase surfaces can be functionalized only within a narrow interval of impact energies. Below 0.016 J, impacts do not seem to have energy enough to induce the surface functionalization of the anatase nanopowder. Conversely, impact energies above 0.032 J induce local conditions severe enough to remove the stearic acid from the anatase surfaces, eventually degrading the surface functionalization even in nanoparticles in which it had been previously attained.

Within the proper range of impact energies, the surface functionalization process displays apparent sigmoidal kinetics. We note that experimental uncertainties are remarkably and inevitably large as a result of the non-homogeneous mechanical processing experienced by the powder mixtures. The apparent kinetic curve can be defined only considering numerous independent samples and characterizing the statistical distribution of the degree of surface functionalization in the different samples.

To interpret the experimental evidence, we developed a kinetic model that accounts for the discontinuous, statistical nature of the mechanical processing by BM. The model combines the statistical factor with the rheology of the powder mixtures and their chemical reactivity. We used the kinetic equation obtained within the model assumptions to best fit the experimental data and separate the three different factors, providing estimates of the rate constants involved.

Interestingly, we observe that the surface functionalization process that takes place on the microscopic scale is characterized by a decreasing reaction probability. This suggests that the presence of molecules of stearic acid bonded to the surface can be associated with a more difficult functionalization of the anatase surface still available to reaction. We have no real possibility to discriminate between a scenario related to the reactivity of surface sites and another one related to steric effects due to molecules already bonded to the surface, except for the experimental evidence, provided by thermograms, that higher temperatures are needed to induce a mass loss at short times of mechanical processing. This suggests that the molecules of stearic acid that react first are also more tightly bound to the surface, which seems reasonable to explain by hypothesizing the existence of surface sites of different reactivity and that more reactive sites react first.

Given the considerations above, we emphasize the capability of the model to shed some light on the chemical behavior of anatase and stearic acid at the molecular level. The model provides a tool to interpret the raw experimental evidence by building a bridge between global and local kinetics. We believe that its systematic use can contribute to building a coherent body of knowledge that can bridge the gap between the macroscopic experimental evidence and the underlying processes that occur on the mesoscopic and microscopic scales.

## Figures and Tables

**Table 1:** The ball mass, $m_{b}$ (g), the coefficient of restitution, e, and the impact energy, E (J), for the BM experiments performed. In all cases, the impact frequency, N, is equal to about 29 Hz.

$m_{b}$	1.0	1.5	2.0	2.5	3.0	3.5	4.0	4.5	5.0	6.0	8.0	12.0
e	0.02	0.02	0.04	0.04	0.05	0.05	0.05	0.05	0.06	0.08	0.10	0.14
E	0.008	0.012	0.016	0.020	0.024	0.028	0.032	0.036	0.039	0.044	0.057	0.078

## Data Availability

The data presented in this study are available within the article.
